# Diethylcarbamazine Attenuates the Development of Carrageenan-Induced Lung Injury in Mice

**DOI:** 10.1155/2014/105120

**Published:** 2014-01-16

**Authors:** Edlene Lima Ribeiro, Karla Patricia de Souza Barbosa, Ingrid Tavares Fragoso, Mariana Aragão Matos Donato, Fabiana Oliveira dos Santos Gomes, Bruna Santos da Silva, Amanda Karolina Soares e Silva, Sura Wanessa Santos Rocha, Valdemiro Amaro da Silva Junior, Christina Alves Peixoto

**Affiliations:** ^1^Pós-Graduação em Ciências Biológicas da Universidade Federal de Pernambuco, Avenida Professor Moraes Rego, s/n, Cidade Universitária, 50670-901 Recife, PE, Brazil; ^2^Instituto Aggeu Magalhães, FIOCRUZ, Avenida Professor Moraes Rego, s/n, Cidade Universitária, 50670-420 Recife, PE, Brazil; ^3^Departamento de Morfologia e Fisiologia Animal da Universidade Federal Rural de Pernambuco, Rua Dom Manoel de Medeiros, s/n, Dois Irmãos, 52171-900 Recife, PE, Brazil; ^4^Laboratório de Ultraestrutura, Instituto Aggeu Magalhães, FIOCRUZ, Avenida Professor Moraes Rego, s/n, Cidade Universitária, 50670-420 Recife, PE, Brazil

## Abstract

Diethylcarbamazine (DEC) is an antifilarial drug with potent anti-inflammatory properties as a result of its interference with the metabolism of arachidonic acid. The aim of the present study was to evaluate the anti-inflammatory activity of DEC in a mouse model of acute inflammation (carrageenan-induced pleurisy). The injection of carrageenan into the pleural cavity induced the accumulation of fluid containing a large number of polymorphonuclear cells (PMNs) as well as infiltration of PMNs in lung tissues and increased production of nitrite and tumor necrosis factor-**α** and increased expression of interleukin-1**β**, cyclooxygenase (COX-2), and inducible nitric oxide synthase. Carrageenan also induced the expression of nuclear factor-**κ**B. The oral administration of DEC (50 mg/Kg) three days prior to the carrageenan challenge led to a significant reduction in all inflammation markers. The present findings demonstrate that DEC is a potential drug for the treatment of acute lung inflammation.

## 1. Introduction

Since 1947, diethylcarbamazine citrate (DEC) has been used in the treatment and control of lymphatic filariasis, which is caused by the nematodes *Wuchereria bancrofti, Brugia malayi, *and* B. timori, *and is one of the drugs used in the Global Programme for the Elimination of Lymphatic Filariasis [1]. However, despite its long period of use, little is known regarding its mechanism of action.

Pharmacological studies have demonstrated that DEC affects the metabolism of arachidonic acid, thereby acting as an anti-inflammatory drug. Substantial evidence has demonstrated that DEC blocks a number of steps in both the cyclooxygenase (COX) and lipoxygenase pathways. This drug is a potent blocker of leukotriene production, and bronchial vasoconstrictor substances and also inhibits the production of prostaglandin (PGE2), prostacyclin (PGI2), and thromboxane A2 (TXA2) [2].

According to Mathews and Murphy (1982) [3], DEC inhibits the formation of LTB_4_ and sulfidopeptide leukotrienes, which are potent vaso/bronchoconstrictors, in mastocytomas, while stimulating the formation of 5-hydroxyeicosatetraenoic acid, suggesting that the site of action of DEC for inhibiting leukotrienes formation may be the leukotriene A_4_ synthetase reaction. Moreover, Bach and Brashler (1986) [4] found that DEC inhibited the formation of sulfidopeptide leukotrienes in rat basophil leukemia cells.

Clinical studies have found that DEC is quite effective in the treatment of symptoms of bronchial asthma [5, 6]. Recent studies carried out in cooperation with our laboratory demonstrated that DEC plays an important role in blocking pulmonary eosinophilic inflammation in mice sensitized with ovalbumin, effectively preventing the effects of subsequent airway resistance, Th1/Th2 cytokine production, pulmonary eosinophil accumulation, and eosinophilopoiesis both *in vivo* and *ex vivo*. Moreover, DEC directly suppressed interleukin-5-dependent eosinophilopoiesis in naive bone marrow [7].

Carrageenan-induced inflammation is a model of local acute inflammation commonly used to evaluate the activity of anti-inflammatory drugs [8] and assess the contribution of cells and mediators to the inflammatory process [9]. The inflammatory process is invariably characterized by the production of prostaglandin, leukotrienes, histamine, bradykinin, platelet-activating factor, interleukins (IL), and migrating cells [10]. The recruitment of polymorphonuclear cells (PMNs) from the circulation to the inflamed tissue has a key function in the breakdown and remodeling of injured tissue [11, 12]. Moreover, macrophages participate in the progression of experimental pleurisy by producing proinflammatory cytokines, such as tumor necrosis factor-*α* (TNF*α*) and IL-1*β* [13]. The initial phase of carrageenan-induced acute inflammation (0 to 1 h) not inhibited by nonsteroidal anti-inflammatory drugs, such as indomethacin, has been attributed to the release of histamine, 5-hydroxytryptamine, and bradykinin, followed by a late phase (1 to 6 h), mainly sustained by prostaglandin release attributed to the induction of cyclooxygenase-2 (COX-2) [13, 14].

Considering the anti-inflammatory properties of DEC as a result of its effect on the metabolism of arachidonic acid, the purpose of the present study was to investigate the anti-inflammatory action of this drug in a model of carrageenan-induced pleurisy (4 h), determining the following end points of the inflammatory response: (1) PMN infiltration; (2) lung injury (histology and ultrastructure); (3) expression of TNF-*α* (ELISA and immunohistochemistry); (4) expression of IL-1*β*, COX-2 (immunohistochemistry and western blot), nitric oxide synthase (iNOS) (immunohistochemistry), and nuclear factor-*κ*B p65 (NFkB p65) (western blot); and (5) the synthesis of nitric oxide (NO) (nitrite concentration).

## 2. Materials and Methods

### 2.1. Animals

Male Swiss mice (weight: 20 to 25 g; CPqAM/PE, Brazil) were used following institutionally approved protocols (CEUA#LW-47/10). The animals were housed in a controlled environment and provided with standard rodent chow and water.

### 2.2. Experimental Groups

Mice were randomly allocated into the following groups:

- sham + water group: in which identical surgical procedures to the carrageenan group (CAR) were performed, but saline was administered instead of carrageenan (intrapleural) (*N* = 10);
- CAR + water group: mice subjected to carrageenan-induced pleurisy (intrapleural) (*N* = 10);
- CAR + DEC group: mice subjected to carrageenan-induced pleurisy and diethylcarbamazine (50 mg/Kg, oral route) three days prior to the carrageenan challenge (*N* = 10);
- CAR + INDO group: mice subjected to carrageenan-induced pleurisy and indomethacin (5 mg/Kg, oral route) three days prior to the carrageenan challenge (*N* = 10).

The therapeutic dose regimen for lymphatic filariasis recommended by the World Health Organization is 6 mg/Kg for 12 days [15]. As the total metabolism rate of mice is approximately seven times that of humans, 50 mg/Kg of DEC was adjusted according to the body weight [16]. The dose of indomethacin (5 mg/Kg) was based on a previous study [17].

### 2.3. Carrageenan-Induced Pleurisy

The mice were anesthetized with an intramuscular injection of a combination of 10% ketamine hydrochloride (115 mg/Kg) and 2% xylazine (10 mg/Kg). After confirmation of analgesia, the right side of the chest was shaven and either sterile saline or sterile saline containing 1% *λ*-carrageenan (0.1 mL) was administered into the pleural cavity in the sixth intercostal space. Four hours after carrageenan injection, the animals were sacrificed through CO_2_ inhalation. The chest was carefully opened and the pleural cavity was rinsed with 1 mL of saline solution containing heparin (5 U/mL) [18]. The exudate and washing solution were removed by aspiration. Any exudate contaminated with blood was discarded. Samples of the fluid from the pleural cavity were collected to determine the total leukocyte content.

Total leukocytes were determined in a Neubauer chamber with the exudate diluted in Turk's solution (1 : 20) [17]. As the carrageenan-induced inflammatory response in the pleural space of mice has a biphasic profile, peaking at 4 and 48 h after pleurisy induction, the expression of inflammatory mediators was measured 4 h after the injection of carrageenan, based on previous studies [8].

### 2.4. Histological Examination

Lung base biopsies were performed 4 h after carrageenan injection. Lung fragments were washed twice in PBS, pH 7.2, and fixed in Bouin's solution (1% saturated picric acid, formaldehyde, and 40% glacial acetic acid) for 8 hours, dehydrated in an increasing ethanol series, cleared in xylene, and embedded in purified paraffin (VETEC, São Paulo, SP, Brazil). Tissue sections of 5 *μ*m were cut using a microtome (Leica RM 2125RT), deparaffinized with xylene, stained with haematoxylin/eosin, and studied using light microscopy [19].

### 2.5. Electron Transmission Microscopy

The lung fragments were fixed overnight in a solution containing 2.5% glutaraldehyde and 4% paraformaldehyde in 0.1 M cacodylate buffer. The samples were then washed twice in the same buffer and postfixed in a solution containing 1% osmium tetroxide, 2 mM calcium chloride, and 0.8% potassium ferricyanide in 0.1 M cacodylate buffer, pH 7.2, dehydrated in acetone, and embedded in Embed 812. Polymerization was performed at 60°C for three days [20]. Ultrathin sections were collected on 300-mesh nickel grids, counterstained with 5% uranyl acetate and lead citrate, and examined using a FEI Morgani 268D transmission electron microscope.

### 2.6. Immunohistochemical Localization of TNF-*α*, IL-1*β*, COX-2, and iNOS

Five sections (5 *μ*m in thickness) from each group were cut and adhered to slides treated with 3-aminopropyltriethoxysilane (APES (Sigma, USA)). Briefly, sections were deparaffinized with xylene and rehydrated in graded ethanol (100 to 70%). To minimize endogenous peroxidase activity, the slides were treated with 10% (v/v) H_2_O_2_ in water for fifteen minutes. The sections were washed with 0.01 M PBS (pH 7.2) and then blocked with 1% BSA, 0.2% Tween 20 in PBS for 1 h at room temperature. The sections were incubated overnight at 4°C with anti-TNF-*α* antibody (ABCAM, CA, USA, 1 : 250), anti-IL-1*β* antibody (GenWay, San Diego, CA, USA, 1 : 250), anti-COX-2 antibody (ABCAM, CA, USA, 1 : 400), and anti-iNOS (ABCAM, CA, USA, 1 : 50). The antigen-antibody reaction was visualized with avidin-biotin peroxidase (Dako Universal LSAB + Kit, Peroxidase), using 3,3-diaminobenzidine as the chromogen. The slides were counterstained with hematoxylin. Positive staining resulted in a brown reaction product. Five pictures at the same magnification were quantitatively analyzed using the Gimp 2.6 software program (GNU Image Manipulation Program, UNIX platforms).

### 2.7. Myeloperoxidase (MPO) Activity

MPO activity, an indicator of PMN accumulation, was determined as previously described [21]. Lung tissues were obtained and weighed, each piece homogenized in a solution containing 0.5% (w/v) hexadecyltrimethylammonium bromide dissolved in 10 mM potassium phosphate buffer (pH 7) and centrifuged for 30 min at 20,000 ×g at 4°C. An aliquot of the supernatant was then allowed to react with a solution of tetramethylbenzidine (1.6 mM) and 0.1 mM hydrogen peroxide. The rate of change in absorbance was measured spectrophotometrically at 450 nm.

### 2.8. Measurement of TNF-*α* Levels

TNF-*α* levels were evaluated in the exudates 4 h after the induction of pleurisy by carrageenan injection. The assay was carried out by using an immunoenzymatic assay, commercial ELISA kit (ABCAM, CA, USA, cat. no. ab100747). The lower detection limit of the assay was 60 pg/mL.

### 2.9. Measurement of NO

The Griess colorimetric reaction was used for the measurement of nitric oxide, involving the detection of nitrite (NO_2_^−^) and the oxidation of NO in the pleural fluid. In duplicate, 50 *μ*L of the pleural fluid was added to a 96-well ELISA plate, followed by the same volume of Griess reagent, which is composed of 1% sulfanilamide diluted in 2.5% H_3_PO_4_ (solution A) and N-1-naphtyl-ethylenediamine also diluted in 2.5% H_3_PO_4_ (solution B). To prepare the standard curve, a solution of sodium nitrite in an initial concentration of 100 *μ*M was serially diluted in PBS. After incubation for 10 minutes in the dark, reading was performed in the spectrophotometer at 490 nm. The absorbance of different samples was compared with the standard curve and the results were expressed as mean ± standard error of the duplicate, using the GraphPad Prism program (v. 5.0) [22].

### 2.10. Western Blot Analysis for COX-2, IL-1*β*, and NF*κ*B

The lungs were quickly dissected and homogenized in a Wheaton Overhead Stirrer (no. 903475) in an extraction cocktail (10 mM ethylenediamine tetraacetic acid (EDTA), 2 mM phenylmethylsulfonyl fluoride (PMSF), 100 mM sodium fluoride (NaF), 10 mM sodium pyrophosphate, 10 mM sodium orthovanadate (NaVO_4_), 10 mg of aprotinin, and 100 mM Tris(hydroxymethyl)aminomethane, pH 7.4). Homogenates were centrifuged at 3000 ×g for 10 min. The supernatant was stored at −70°C until use for immunoblotting. Protein levels were determined by the Bradford method using bovine serum albumin as the standard [23]. The proteins (40 *μ*g/*μ*L) were separated on 10% (COX-2 and NF*κ*B) or 14% (IL-1*β*) sodium dodecyl sulfate polyacrylamide by gel electrophoresis under reduced conditions and were electrophoretically transferred onto the nitrocellulose membrane (Bio Rad, CA, USA, Ref. 162-0115). After blocking overnight at 4°C with 5% nonfat milk in TBS-T (Tris-buffered saline 0.1% plus 0.05% Tween 20, pH 7.4), the membranes were incubated at room temperature for 3 h with rabbit polyclonal antibody against COX-2 (1 : 1,000 dilution; ABCAM, CA, USA), IL-1*β* (1 : 2,000 dilution, Genway, San Diego, CA, USA) and NF*κ*B (1 : 200 dilution, Santa Cruz Biotechnology, Santa Cruz, CA, USA) diluted in buffer solution TBS-T containing 3% nonfat milk. After washing six times (10 min each) in TBS-T, the membranes were further reacted with horseradish peroxidase-conjugated anti-rabbit or anti-mouse secondary antibody (1 : 80,000 (Ref. A6154) and 1 : 80,000 (Ref. A5420), respectively; Sigma, USA) diluted in TBS-T with 1% nonfat milk for 1 h 30 min at room temperature. An enhanced chemiluminescence reagent (Super Signal, Pierce, Ref. 34080) was used to make the labeled protein bands visible and the blots were developed on X-ray film (Fuji Medical, Kodak, Ref. Z358487-50EA). For quantification, the density of pixels of each band was determined using the ImageJ 1.38 program (available at http://rsbweb.nih.gov/ij/download.html; developed by Wayne Rasband, NIH, Bethesda, MD, USA). For each protein investigated, the results were confirmed in three sets of experiments. Immunoblotting for *β*-actin was performed as a control for the above protein blots.

After the visualization of the protein blots with enhanced chemiluminescence, the protein antibodies were stripped from the membranes, which were reprobed with monoclonal anti-*β*-actin antibody (1 : 2,000 dilution, Sigma, USA), and protein densitometry was subsequently performed.

### 2.11. Data Analysis

All values are expressed as mean and standard error of the mean (±S.E.M.) of *n* observations. In the *in vivo* studies, *n* represents the number of animals studied. The results were analyzed by one-way ANOVA followed by Tukey's posttest, using the GraphPad Prism program (V. 5.0). All *P* values less than 0.05 were considered significant.

## 3. Results

### 3.1. Effect of DEC on Leukocyte Migration

**Figure 1:**
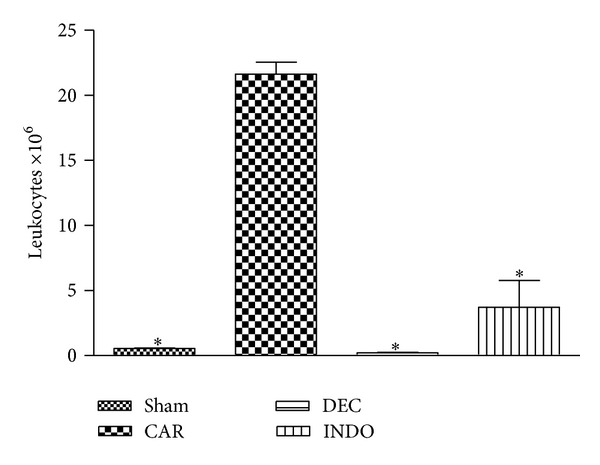
Effect of diethylcarbamazine (DEC: 50 mg/Kg, three days before) on cell migration in the initial phase (4 h) of the inflammatory reaction induced by carrageenan in mice; data expressed as mean ± S.E.M of 10 mice for each group; *P < 0.05 versus carrageenan.

The injection of carrageenan into the pleural cavity of mice induced an acute inflammatory response characterized by the accumulation of fluid containing a large amount of PMNs. However, the number of PMNs was significantly reduced with prior treatment for three days with DEC and INDO (0.22 ± 0.09 × 10^6^ and 3.72 ± 5.05 × 10^6^ leukocytes, resp.) compared to the group that received carrageenan without prior treatment (21.63 ± 2.27 × 10^6^ leukocytes) (Figure 1).

### 3.2. Effect of Diethylcarbamazine on Carrageenan-Induced Tissue Damage

**Figure 2:**
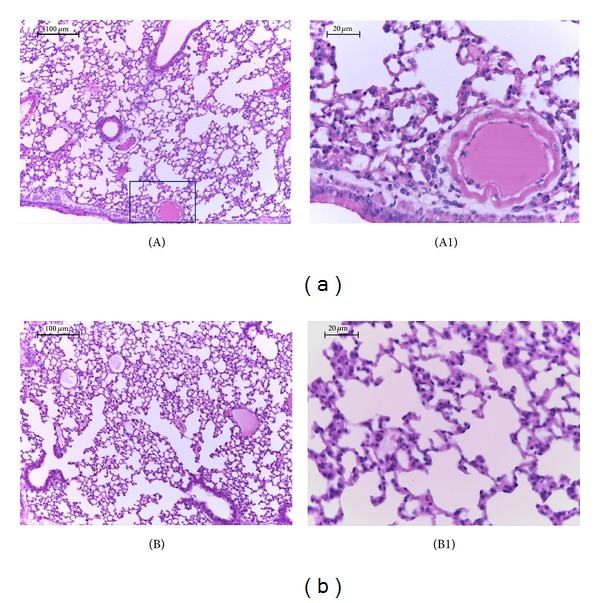
Effect of DEC treatment on histological alterations in lung after carrageenan-induced injury; (A)-(A1) lung sections taken from mice with carrageenan-induced pleurisy demonstrated tissue injury as evidenced in edema, cellularity enhancement, and polymorphonuclear infiltration. (B)-(B1) Treatment with DEC three days prior to pleurisy demonstrated reduced lung injury and PMN infiltration. The figure is representative of at least 3 experiments performed on different experimental days. *n* = 10 mice for each group; scale bar = 100 *μ*m and 20 *μ*m.

The histological analysis revealed that the animals with carrageenan-induced pleurisy exhibited discrete alveolar thickening due to increased cellularity, mild hemorrhage and congestion, apoptotic cells, inflammatory cells (mononuclear and polymorphonuclear cells), and pulmonary edema and emphysema (Figure 2(a)). Treatment for three days with DEC attenuated the degree of injury and the infiltration of PMNs (Figure 2(b)). The lungs in the sham group exhibited preserved morphological characteristics.

**Figure 3:**
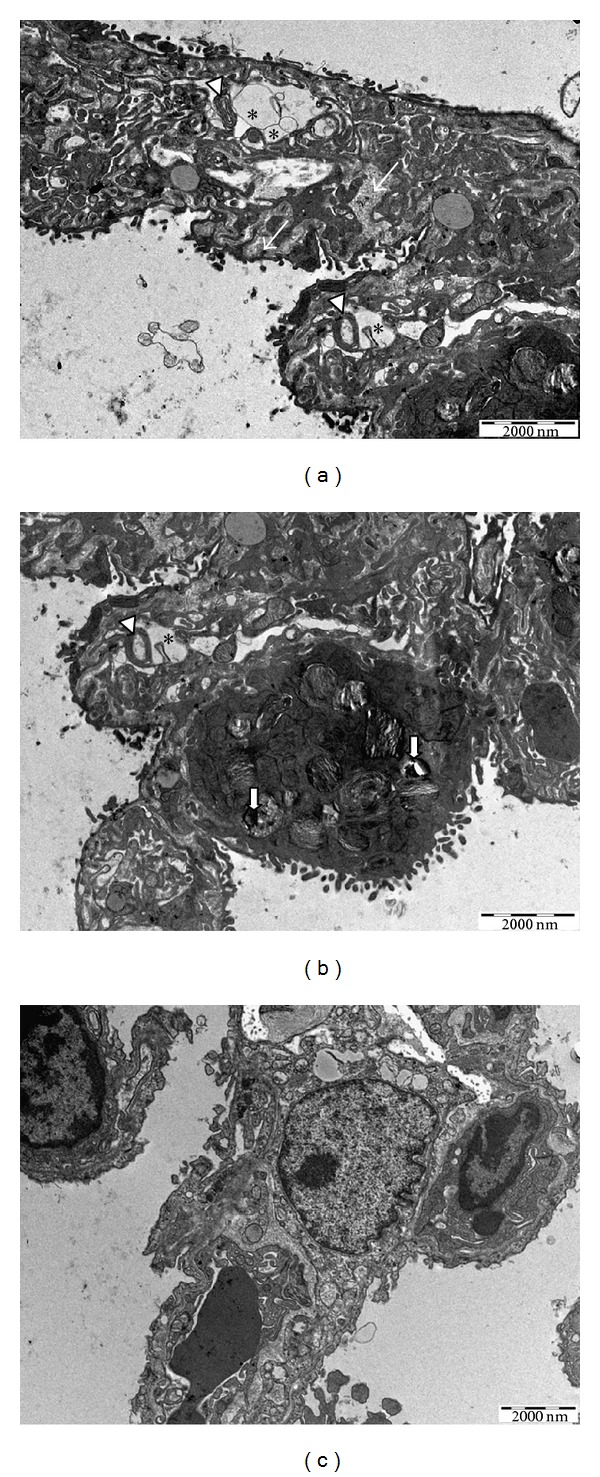
Ultrastructural analysis of lung after carrageenan-induced injury and DEC treatment; (a) and (b) lung sections from mice with carrageenan-induced pleurisy showing enhanced thickness of the interstitial space filled with collagen fibers (thin arrows), myelin bodies (arrowheads), vacuoles (asterisks), and lamellar bodies containing electrodense granules (short arrows); (c) lung treated with DEC presenting preserved pneumocytes; bar = 2000 nm.

The pulmonary ultrastructural analysis of the animals in the sham group revealed a preserved morphological pattern, such as respiratory spaces, including the alveolar epithelium composed of pneumocytes (not shown). The lung tissue of animals with carrageenan-induced pleurisy revealed type II pneumocytes with lamellar bodies containing electrodense granules, vacuoles, and myelin bodies, characterizing cell suffering. Numerous collagen fibers were also observed in the interstitial space, increasing its thickness (Figures 3(a) and 3(b)). The animals treated with DEC exhibited a preserved alveolar epithelium similar to that found in the sham group (Figure 3(c)).

### 3.3. Effect of DEC on MPO Activity in Mice

**Figure 4:**
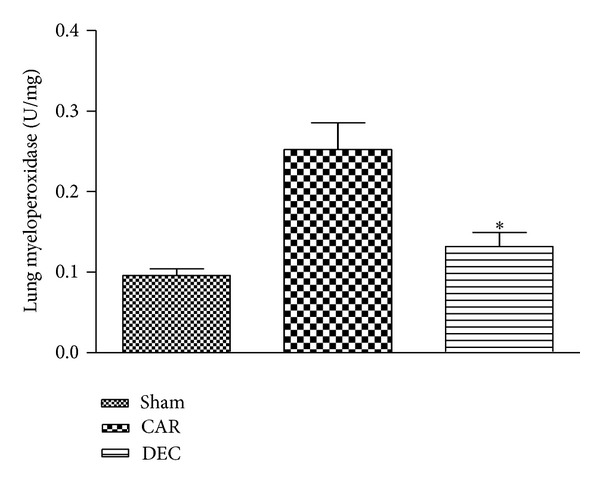
Within 4 h, pleural injection of carrageenan led to an increase in neutrophil accumulation in the lung. DEC treatment significantly inhibited neutrophil infiltration. Data expressed as mean ± S.E.M. from *n* = 8 mice for each group; *P < 0.05 versus carrageenan.

The pleural infiltration with PMN appeared to correlate with an influx of leukocytes into the lung tissue; thus we investigated the effect of DEC on neutrophil infiltration by measurement of MPO activity. This enzyme activity was significantly elevated at 4 h after CAR administration. Treatment with DEC significantly attenuated a neutrophil infiltration into the lung tissue (Figure 4).

### 3.4. Effect of DEC on Expression of TNF-*α*, IL-1*β*, COX-2, and iNOS

**Figure 5:**
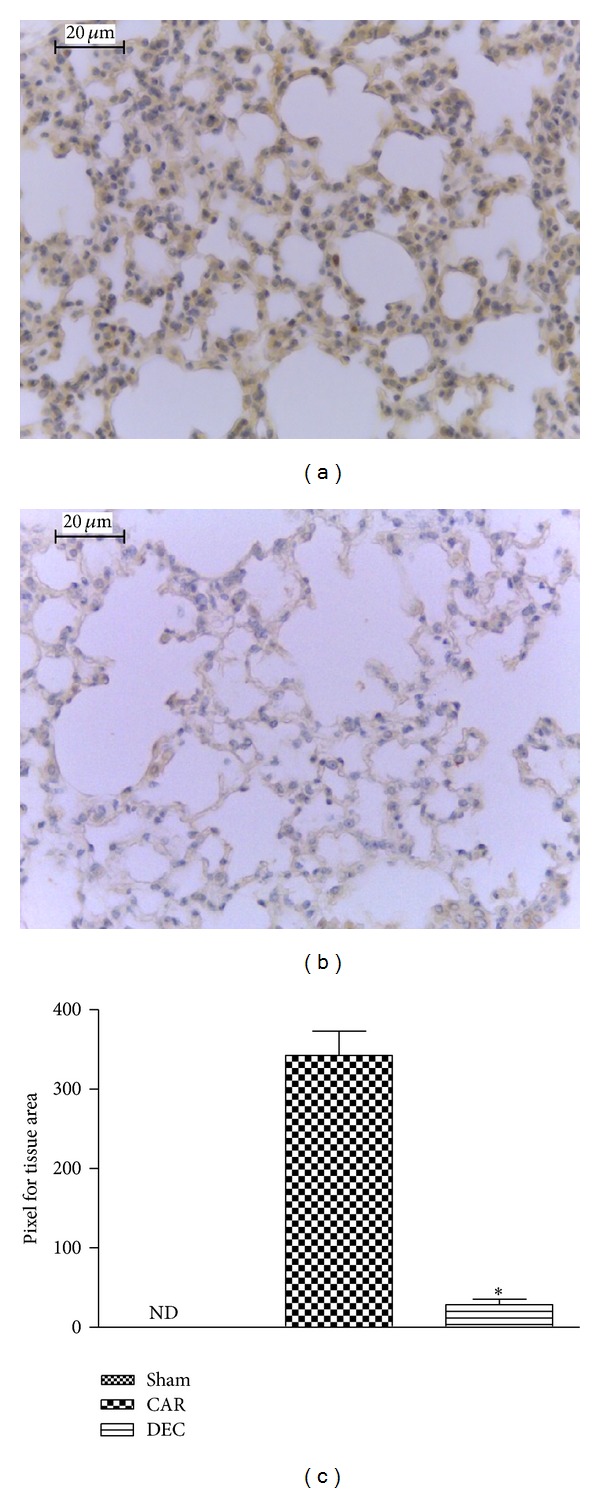
Effect of DEC on immunohistochemical localization TNF-*α* in lung after carrageenan-induced pleurisy. (a) In tissue sections obtained from the CAR group, positive staining for TNF-*α* was mainly located in inflammatory cells. (b) After treatment with DEC, the degree of positive staining for TNF-*α* was reduced in the lung tissue. (c) Densitometry analysis of immunohistochemistry photographs for TNF-*α* in lung tissues; figure representative of at least 3 experiments performed on different experimental days; data expressed as mean ± S.E.M. from *n* = 5 mice for each group; ND: not detected; *P < 0.05 versus carrageenan; scale bar = 20 *μ*m.

The expression of TNF-*α*, IL-1*β*, COX-2, and iNOS in the lung tissue was evaluated by immunohistochemical detection. Tissue sections obtained from mice in the CAR group had positive staining for TNF-*α* in the alveolar cells, macrophages, and vascular wall (Figure 5(a); densitometry analysis: Figure 5(c)). In contrast, no staining for TNF-*α* was found in the lungs of mice treated with DEC (Figure 5(b); densitometry analysis: Figure 5(c)). Likewise, no positive staining for TNF-*α* was found in lung tissue from the sham-treated mice.

**Figure 6:**
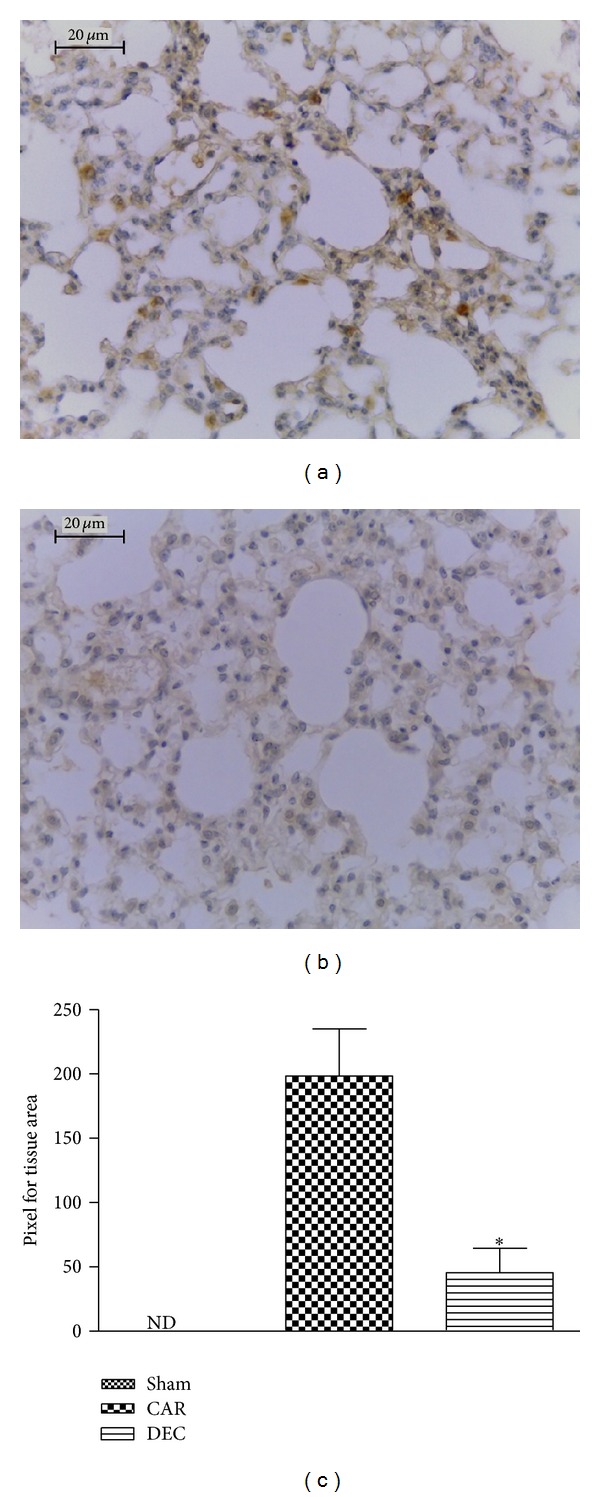
Effects of DEC on immunohistochemical localization of IL-1*β*. (a) At 4 h after carrageenan injection, staining intensity for IL-1*β* substantially increased in alveolar macrophages. (b) No positive staining for IL-1 was found when DEC was administered three days prior to carrageenan injection. (c) Densitometry analysis of immunohistochemistry photographs for IL-1-*β* in lung tissues; figure representative of at least 3 experiments performed on different experimental days; data expressed as mean ± S.E.M. from *n* = 5 mice for each group; ND: not detected; *P < 0.05 versus carrageenan; scale bar = 20 *μ*m.

Lung tissue sections from mice in the CAR group demonstrated a positive reaction for IL-1*β* in alveolar macrophages (Figure 6(a); densitometry analysis: Figure 6(c)). Treatment with DEC for three days significantly reduced the degree of IL-1*β* expression (Figure 6(b); densitometry analysis: Figure 6(c)). There was no labeling for IL-1*β* in lung tissue obtained from mice in the sham group.

**Figure 7:**
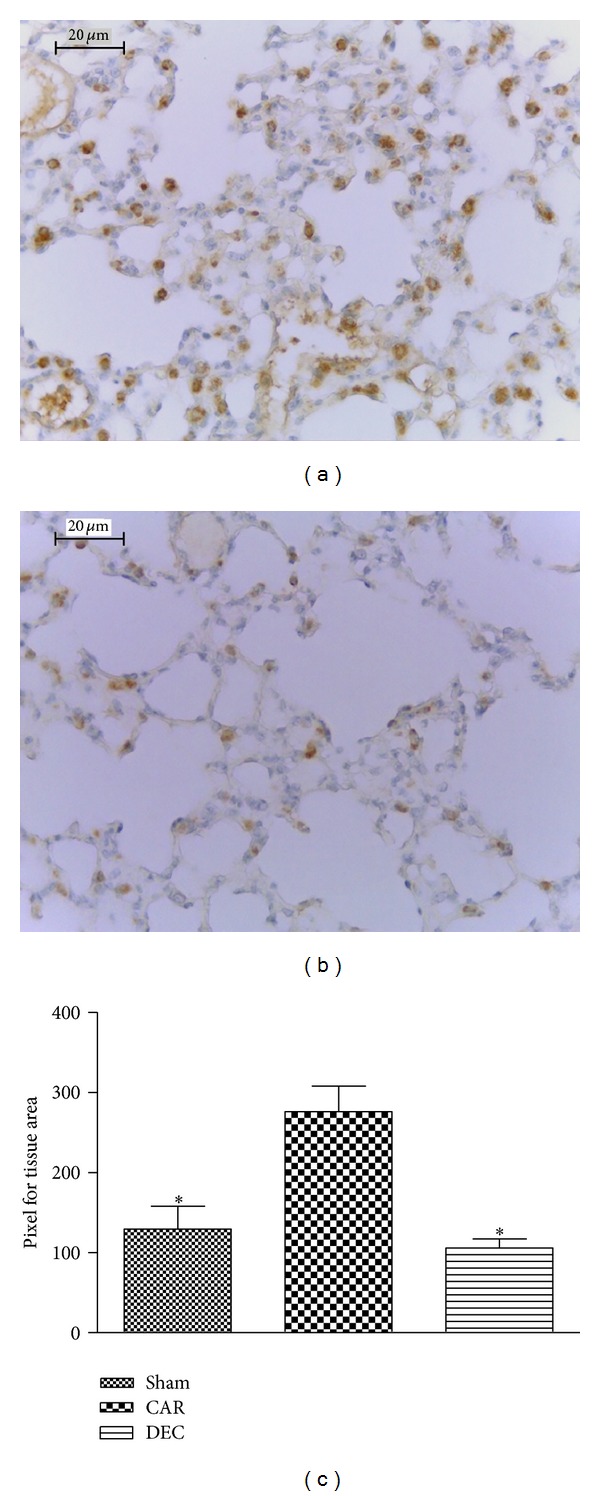
Effect of DEC on immunohistochemical localization of COX-2 in lung tissue after pleurisy induced by carrageenan. (a) In tissue sections from the CAR group, positive labeling was detected on type II pneumocytes. (b) Treatment with DEC significantly reduced COX-2 staining in comparison to the CAR group, achieving levels similar to the sham group. (c) Densitometry analysis of immunohistochemistry photographs for COX-2 from lung tissues; figure is representative of at least 3 experiments performed on different experimental days; data expressed as mean ± S.E.M. from *n* = 5 mice for each group; ND: not detected; *P < 0.05 versus carrageenan; scale bar = 20 *μ*m.

The lung tissue sections obtained from mice in the CAR group revealed considerable COX-2 expression (Figure 7(a); densitometry analysis Figure 7(c)), whereas COX-2 expression was significantly reduced in lung sections obtained from mice treated with DEC (Figure 7(b); densitometry analysis: Figure 7(c)). Lung sections from the mice in the sham group expressed baseline levels of COX-2.

**Figure 8:**
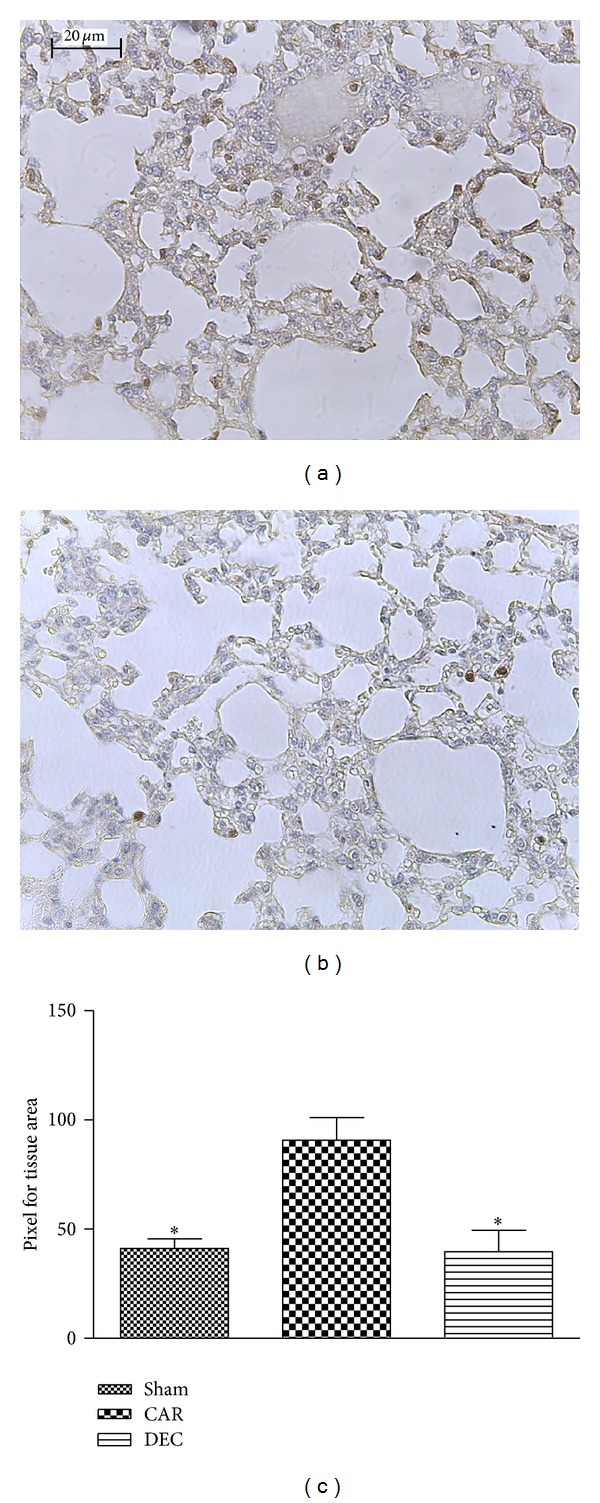
Effect of DEC on immunohistochemical localization of iNOS in lung tissue after pleurisy induced by carrageenan. (a) In tissue sections from the CAR group, positive labeling was detected on alveolar macrophages. (b) Treatment with DEC significantly reduced the iNOS staining in comparison to the CAR group, achieving levels similar to the sham group. (c) Densitometry analysis of immunohistochemistry photographs for iNOS from lung tissues; figure representative of at least 3 experiments performed on different experimental days; data expressed as mean ± S.E.M. from *n* = 5 mice for each group; *P < 0.05 versus carrageenan; scale bar = 20 *μ*m.

Lung sections obtained from mice in the CAR group revealed positive staining for iNOS in alveolar macrophages (Figure 8(a); densitometry analysis: Figure 8(c)), whereas DEC treatment significantly attenuated iNOS expression (Figure 8(b); densitometry analysis: 8C). Little staining for iNOS was observed in the lung tissue obtained from the sham group.

### 3.5. Effect of DEC on TNF-*α* and NO Concentration in Pleural Exudate

**Figure 9:**
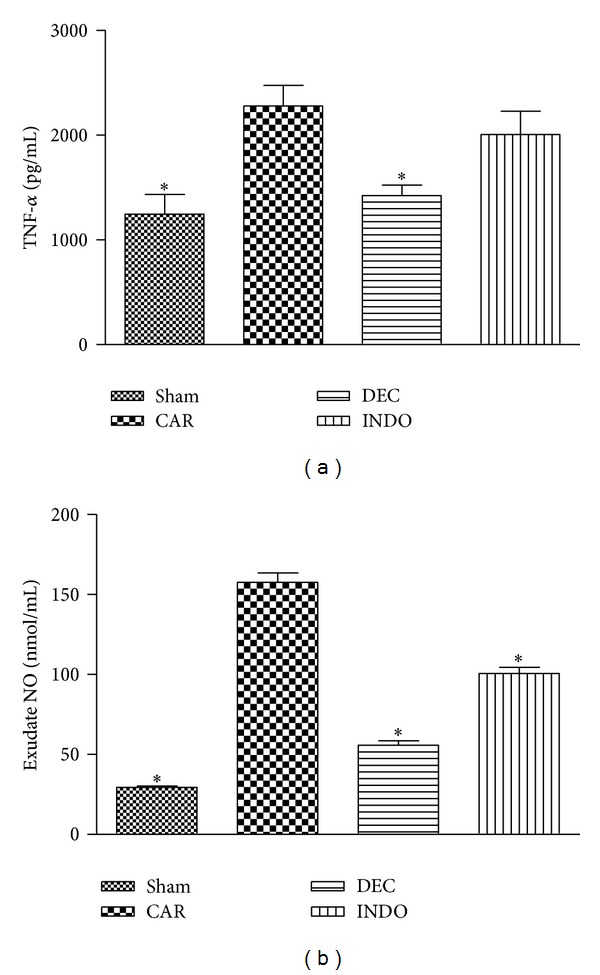
Effect of DEC on carrageenan-induced TNF-*α* and NO production in the lung. (a) TNF-*α* levels were significantly elevated 4 h after carrageenan administration in the CAR group in comparison to the sham group. DEC significantly reduced the TNF-*α* level, but INDO did not reduce TNF-*α* level in comparison to the CAR group. Nitrite and nitrate levels, stable NO metabolites, were significantly increased in the pleural exudates 4 h after carrageenan administration in comparison to the sham group. DEC and INDO significantly reduced the nitrite and nitrate level in the exudates (b). Data expressed as mean ± S.E.M. from *n* = 8 mice for each group; *P < 0.05 versus carrageenan.

The concentration of TNF-*α* in the pleural exudate was analyzed using enzyme-linked immunosorbent assays (ELISA). Carrageenan-induced pleurisy promoted high levels of TNF-*α* in the pleural exudate in comparison to the sham group, whereas treatment with DEC for three days prior to the induction of pleurisy significantly attenuated the production of TNF-*α*. In contrast, treatment with indomethacin promoted no reduction in the level of this proinflammatory cytokine (Figure 9(a)).

NO in the pleural exudate were analyzed through the Griess reaction. NO levels increased significantly in the exudate in the CAR group in comparison to the sham group. However, treatment with DEC and INDO significantly reduced NO levels in comparison to the CAR group (Figure 9(b)).

### 3.6. Western Blot Analysis for COX-2, IL-1*β*, and NF*κ*B

**Figure 10:**
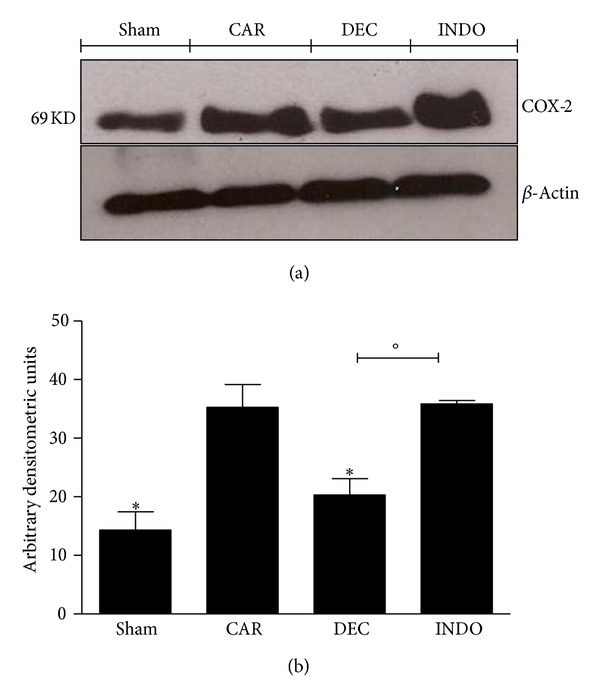
Effects of DEC on carrageenan-induced COX-2 expression in the lung. Basal expression of COX-2 was detected in lung samples from the sham group, whereas COX-2 levels were substantially elevated in lung tissue obtained from animals 4 h after carrageenan injection. DEC treatment reduced the expression of COX-2, but treatment with indomethacin did not decrease the levels of COX-2 (a) and (b). (a) Representative blot of lysates obtained from pool 4 animals per group; (b) data expressed as mean ± S.E.M. of 4 replications for each group; *P < 0.05 versus carrageenan; °*P* < 0.05 versus DEC.

The presence of COX-2 in the lung homogenate was investigated by western blot analysis 4 hours after the induction of pleurisy. Baseline levels of COX-2 were detected in the mice in the sham group, whereas COX-2 levels were significantly increased in lung tissue from the CAR group. However, treatment with DEC significantly reduced the expression of COX-2. Unexpectedly, treatment with indomethacin did not reduce the levels of COX-2 in comparison to the CAR group (Figure 10).

**Figure 11:**
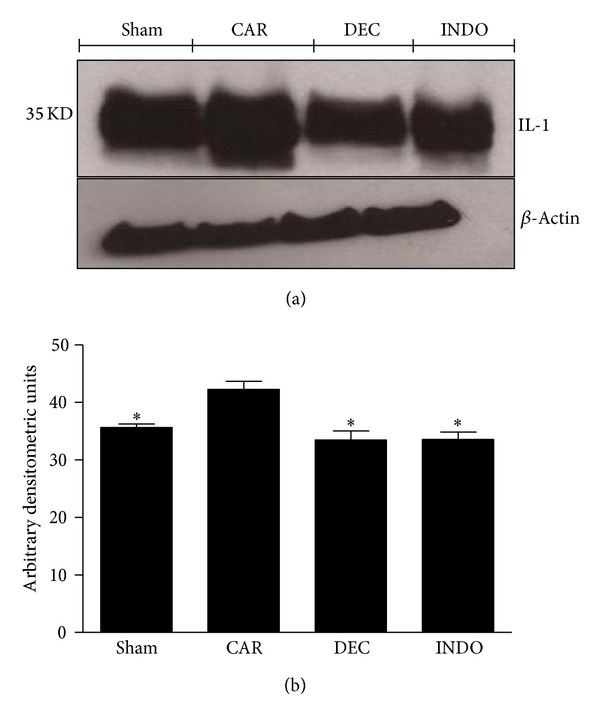
Effects of DEC on carrageenan-induced IL-1*β* expression in the lung. Basal expression of IL-1*β* was detected in lung samples obtained from the sham group, whereas levels of IL-1*β* were significantly increased in the lung tissue from animals 4 hours after injection of carrageenan. Treatment with DEC and INDO reduced the expression of IL-1*β* in comparison to the CAR group. (a) Representative blot of lysates obtained from pool 4 animals per group; (b) data expressed as mean ± S.E.M. of 3 replications for each group; *P < 0.05 versus carrageenan.

Baseline levels of IL-1*β* expression were detected in the lung homogenate from the sham group, whereas increased levels were found in the CAR group. Treatment with DEC and INDO decreased significantly the IL-1*β* levels in comparison to the CAR group (Figure 11).

**Figure 12:**
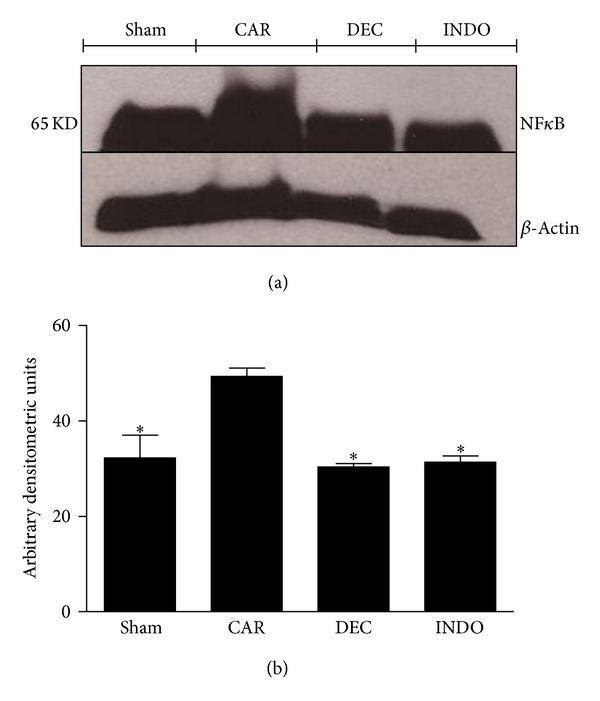
Effects of DEC on carrageenan-induced NF*κ*B expression in the lung. Basal expression of NF*κ*B was detected in lung samples obtained from the sham group, whereas NF*κ*B levels were significantly increased in lung tissue of animals 4 hours after injection of carrageenan. Treatment with DEC and INDO reduced NF*κ*B expression in comparison to the CAR group. (a) Representative blot of lysates obtained from pool 4 animals per group; (b) data expressed as mean ± S.E.M. of 3 replications for each group; *P < 0.05 versus carrageenan.

NF*κ*B p65 levels in the lung were also significantly increased at 4 h after carrageenan injection in comparison to the sham group, whereas DEC and INDO both significantly decreased the NF*κ*B levels in comparison to the CAR group (Figure 12).

## 4. Discussion 

Acute pulmonary inflammation is associated with the enhanced formation of the proinflammatory cytokines TNF-*α* and IL-1*β*, inducible COX-2, and the production of reactive oxygen species (ROS), such as hydrogen peroxide, superoxide, and hydroxyl radicals [9, 24]. In the present study, the injection of carrageenan into the pleural cavity induced PMNs infiltration, lung injury (characterized by cellular infiltration, edema, alveolar thickness, myelin bodies, and large vacuoles), and the production of proinflammatory cytokines as well as COX-2 and NOS. Moreover, the histological and ultrastructural analyses demonstrated that DEC efficiently blocked carrageenan-induced lung injury.

Oxidative stress has been shown to play a critical role in acute and chronic inflammatory responses, such as lung injury. The activation of leukocytes, such as neutrophils, prior to the cell responses involved in the acute inflammatory process promotes the release of several types of ROS [17]. NO synthesized by iNOS is another free radical present during inflammation and capable of interacting with ROS to increase the action of free radicals [25]. These radicals are released by various cell types in response to stimulation by TNF-*α* and IL-1*β*, all of which activate a cytoplasmic form of the transcription factor NF-kappaB by releasing an inhibitory protein subunit [26].

Experimental evidence has clearly suggested that NF-*κ*B plays a central role in the regulation of a large number of genes responsible for the generation of mediators or proteins in carrageenan-induced acute lung inflammation, such as TNF-*α*, IL-1*β*, iNOS, and COX-2 [8]. The present findings demonstrate that the inflammatory process caused by the injection of carrageenan into the pleural cavity leads to a substantial increase in levels of TNF-*α* and IL-1*β* in the exudate and lung tissue. Therefore, the inhibition of the release of TNF-*α* and IL-1*β* by DEC described in the present study could be attributed to the inhibitory effects of the activation of NF-*κ*B. However, further studies are needed to clarify the signal transduction pathways involved.

In human lungs, neutrophils, eosinophils, macrophages, platelets, and airway epithelial cells have been described as the main cellular sources of lipoxygenase-derived arachidonic acid products and DEC has been used as a potent lipoxygenase inhibitor of alveolar macrophages by blocking the release of chemotactic activity [27]. Moreover, Stenmark et al. [28] found that DEC blocks the lipoxygenase pathway in the pathogenesis of pulmonary hypertension induced by monocrotaline in mice. Lipoxygenase products are potent inflammatory agents that induce vascular permeability and bronchoconstriction. According to the authors cited, treatment with DEC improved systolic blood pressure and heart weight, reduced the number of PMNs in the bronchoalveolar lavage fluid, and decreased levels of prostaglandins and thromboxane.

Carrageenan-induced pleurisy is a well-characterized experimental model of inflammation used to evaluate cell migration and other inflammatory parameters. Nonsteroidal anti-inflammatory drugs are effective in inhibiting both cell migration and exudation [29]. Based on the present results, the injection of carrageenan into the pleural cavity induced PMNs infiltration, but treatment with DEC significantly reduced the number of leucocytes in the exudate.

Using a carrageenan-induced model of acute inflammation, Tomlinson et al. [9] demonstrated that the influx of PMNs increases the production of COX-2 and iNOS following the induction of pleurisy, as the inflammation progressed and the cell population changed from the PMN to mononuclear profile and there was a decrease in COX and NOS activity. The authors suggest that the use of selective inhibitors of COX-2 and iNOS in acute inflammation may be more beneficial than existing therapies. In general, iNOS-derived NO and COX-2-derived PGs are involved in both acute and chronic inflammation [30].

There is ample evidence in carrageenan and other models of inflammation that the enhanced formation of prostanoids following the induction of COX-2 contributes to the pathophysiology of local inflammation [31, 32] and that selective inhibitors of COX-2 exert potent anti-inflammatory effects. The present results demonstrate that treatment with DEC significantly decreased the levels of COX-2 in lung tissue, as observed with other nonsteroidal anti-inflammatory drugs. DEC inhibits platelet aggregation, possibly due to its effects on the COX pathway [2], which has similarities with the NO pathway, since both have constitutive and inducible isoforms of their enzymes and are key regulators of inflammatory responses [33, 34].

In a study with knockout mice for the iNOS gene (iNOS −/−), Mcgarry et al. [35] demonstrated that nitric oxide synthase (iNOS) pathways likely exert an effect on DEC activity through the interaction with the cyclooxygenase. The authors found that DEC had no microfilaricidal activity in iNOS-deficient mice infected with *B. malayi* and there was a remarkable reduction in the COX-1 protein in the peritoneal exudate. Therefore, the iNOS/COX pathway appears to be an essential event in the rapid sequestration of microfilariae following treatment with DEC.

Queto et al. [7] demonstrated that DEC has an important action in blocking eosinophilic lung inflammation in mice sensitized with ovalbumin. Treatment with DEC reduced the amount of eosinophils in bronchoalveolar fluid and tissue infiltrate and altered the generation of cytokines involved in the production, activation, and migration of eosinophils. The authors provide the first evidence of the therapeutic mechanism of DEC in a model eosinophilic lung inflammation. Interestingly, DEC blocks pulmonary hyperreactivity, Th2 cytokine production, and the accumulation of eosinophils as well as eosinophilopoiesis both *in vivo* and *in vitro* through iNOS/CD95L mechanisms.

Inhibitors of NOS activity reduce the development of carrageenan-induced inflammation and indicate a role for NO in the pathophysiology associated with this inflammation model [36, 37]. The present results demonstrate the inhibition of NOS activity following treatment with DEC, since the formation of nitrite was evidently reduced in the pleural exudates.

In conclusion, the present findings demonstrate for the first time that the administration of DEC in a model of acute inflammation induced by carrageenan led to reductions in lung injury, PMNs migration, the formation of NO production, and the release of proinflammatory cytokines and COX-2, thereby confirming previous observations that DEC effectively acts through the NOS/COX mechanism.
